# Too hot, too cold, or just right: Can wildfire restore dry forests of the interior Pacific Northwest?

**DOI:** 10.1371/journal.pone.0281927

**Published:** 2023-02-27

**Authors:** Skye M. Greenler, Christopher J. Dunn, James D. Johnston, Matthew J. Reilly, Andrew G. Merschel, R. Keala Hagmann, John D. Bailey

**Affiliations:** 1 Department of Forest Engineering, Resources, and Management, Oregon State University, Corvallis, Oregon, United States of America; 2 Department of Forest Ecosystems and Society, Oregon State University, Corvallis, Oregon, United States of America; 3 Pacific Northwest Research Station, United States Department of Agriculture, United States Forest Service, Corvallis, Oregon, United States of America; 4 School of Environmental and Forest Sciences, University of Washington, Seattle, Washington, United States of America; 5 Applegate Forestry LLC, Corvallis, Oregon, United States of America; Weyerhaeuser Company, UNITED STATES

## Abstract

As contemporary wildfire activity intensifies across the western United States, there is increasing recognition that a variety of forest management activities are necessary to restore ecosystem function and reduce wildfire hazard in dry forests. However, the pace and scale of current, active forest management is insufficient to address restoration needs. Managed wildfire and landscape-scale prescribed burns hold potential to achieve broad-scale goals but may not achieve desired outcomes where fire severity is too high or too low. To explore the potential for fire alone to restore dry forests, we developed a novel method to predict the range of fire severities most likely to restore historical forest basal area, density, and species composition in forests across eastern Oregon. First, we developed probabilistic tree mortality models for 24 species based on tree characteristics and remotely sensed fire severity from burned field plots. We applied these estimates to unburned stands in four national forests to predict post-fire conditions using multi-scale modeling in a Monte Carlo framework. We compared these results to historical reconstructions to identify fire severities with the highest restoration potential. Generally, we found basal area and density targets could be achieved by a relatively narrow range of moderate-severity fire (roughly 365–560 RdNBR). However, single fire events did not restore species composition in forests that were historically maintained by frequent, low-severity fire. Restorative fire severity ranges for stand basal area and density were strikingly similar for ponderosa pine (*Pinus ponderosa*) and dry mixed-conifer forests across a broad geographic range, in part due to relatively high fire tolerance of large grand (*Abies grandis*) and white fir (*Abies concolor)*. Our results suggest historical forest conditions created by recurrent fire are not readily restored by single fires and landscapes have likely passed thresholds that preclude the effectiveness of managed wildfire alone as a restoration tool.

## Introduction

Wildfire is a foundational disturbance that has shaped ecosystems for millennia, but the impacts of contemporary wildfires in the western United States have become an increasing social, economic, and ecological concern [1–4]. Aggressive fire exclusion policies, Euro-American colonization, forest and resource management practices, and anthropogenic climate change have altered forest structure and composition in many western US forests and increased vulnerability to uncharacteristically extreme wildfires and drought [5–10]. In many systems, fuel reduction treatments, including mechanical thinning and prescribed fire, can reduce community and ecosystem risk but the pace and scale of treatments are far below that which would substantially alter fire effects and behavior across most landscapes [11–15]. Given limited resources and extensive treatment backlog, there is increasing consideration of wildfire, landscape-scale prescribed fire, and cultural burning as suitable pathways towards wildfire risk reduction and restoration of fire-prone landscapes [16–20].

In the last several years there has been increased effort to identify and address barriers to managed wildfire, large-scale prescribed fire, and cultural burning at broad spatial scales [14, 21–23]. In the western US, work is underway to better align ecological values, operational fire management considerations, and societal values to provide opportunities for managed wildfire [24–27]. Tribes across the US are actively working to address barriers to Indigenous fire stewardship and restore forests that were tended with extensive and repeated cultural burning [22, 28, 29]. While these efforts are building opportunity for managed wildfire, prescribed fire use, and cultural burning, there is still substantial uncertainty about the extent to which fires can achieve desired outcomes [14, 30].

Research in landscapes that have been allowed to burn with minimal human intervention for the last 30–50 years suggests that repeated and interacting fires can promote development of wildfire adapted forest conditions and confer resistance to contemporary wildfires [31–36]. However, in the vast majority of fire-prone western landscapes, forest structure and composition are no longer resistant or resilient to natural disturbance processes, such as interactions between wildfire, endemic insects and pathogens, and drought [1, 37–41]. Given the disconnect between contemporary landscape conditions and native disturbance agents and processes, the degree to which contemporary first-entry wildfires (initial burns after decades of fire exclusion, *sensu* [42]) restore fire resilient structure and composition is not well understood.

For many decades fire ecologists, forest managers, and the public have been primarily focused on high-severity fire effects—where fires burned ‘too hot’ [9, 43]. Recently, fire refugia, unburned or low-severity areas within a fire footprint, have received increased attention as a counterpart to high-severity patches [44–46]. However, research suggests that moderate-severity fire occurring between these two extremes may promote forest structure most similar to fire resistant historical conditions and help achieve restoration and risk reduction goals [20, 42, 47–49]. Although contemporary wildfires increasingly burn at uncharacteristically high severities or extents, the majority of the area burned in the western US is still low- or moderate-severity [1, 6, 50, 51]. A recent analysis of fires across Oregon and Washington, found that 45–54% of burned area from 1985–2010 was low severity in systems characterized historically by low- and mixed-severity regimes [6]. These low severity effects may be ‘too cold’ to achieve restoration objectives in areas where significant tree density reduction or tree species compositional shifts are required [6, 52, 53]. Honing our understanding of the fire severities that are the most restorative requires empirical modeling that can be applied beyond individual fire events across a broad range of forest and biophysical conditions.

Predictive models that estimate fire-induced tree mortality are typically based on relationships between observed or estimated metrics of fire injury to individual trees (e.g., flame length, scorch height, crown scorch) and tree characteristics (e.g., species, diameter, height) [54, 55]. These methods provide reasonable estimates of individual tree mortality for planning small to moderate scale projects, including prescribed fires or salvage harvests, but are rarely used to continuously predict fire-induced mortality across large landscapes given the quantity and quality of data required to produce reliable estimates of flame length or scorch height [55–57]. Instead, after wildfires, managers and scientists often rely on remotely sensed estimates of changes in spectral reflectance (such as the relativized difference normalized burn index, RdNBR) to assess broad-scale patterns of tree mortality [58]. However, evidence suggests that relationships between probability of tree mortality and fire severity, as estimated by RdNBR, vary between tree species and size classes [34, 47, 59–61].

To evaluate the role of contemporary fires in achieving restoration goals, landscape-scale measurements of vegetation change paired with estimates of post-fire tree species composition and structure are needed. Although tools exist to estimate species-specific tree mortality from flame length, fireline intensity, and tree demography (e.g., FVS-FFE, BehavePlus, or FOFEM), few exist to estimate tree mortality from remotely sensed metrics of fire severity (e.g., Monitoring Tends in Burn Severity, www.mtbs.gov). This scale mismatch means that one of the most frequently used estimates of fire severity, RdNBR, does not describe the composition or structure of the surviving forest, which is a critical component of many restoration objectives and strongly influences post-fire regeneration dynamics, vulnerability to drought and future fires, and habitat quality for species of management concern [62–66].

In this study, we used a multi-step modeling process to assess the restoration potential of fire in dry conifer forests of the interior Pacific Northwest using RdNBR. Our objectives for this work were: 1) identify the fire severity range most likely to restore historical conditions in dry mixed-conifer and ponderosa pine (*Pinus ponderosa*) forests across eastern Oregon, and 2) assess uncertainty of modeled mortality estimates at the tree, species, and stand levels. We conclude by demonstrating the application of these methods on a case study landscape and discuss the potential for using remotely sensed severity values to estimate restoration potential.

## Methods

**Fig 1:**
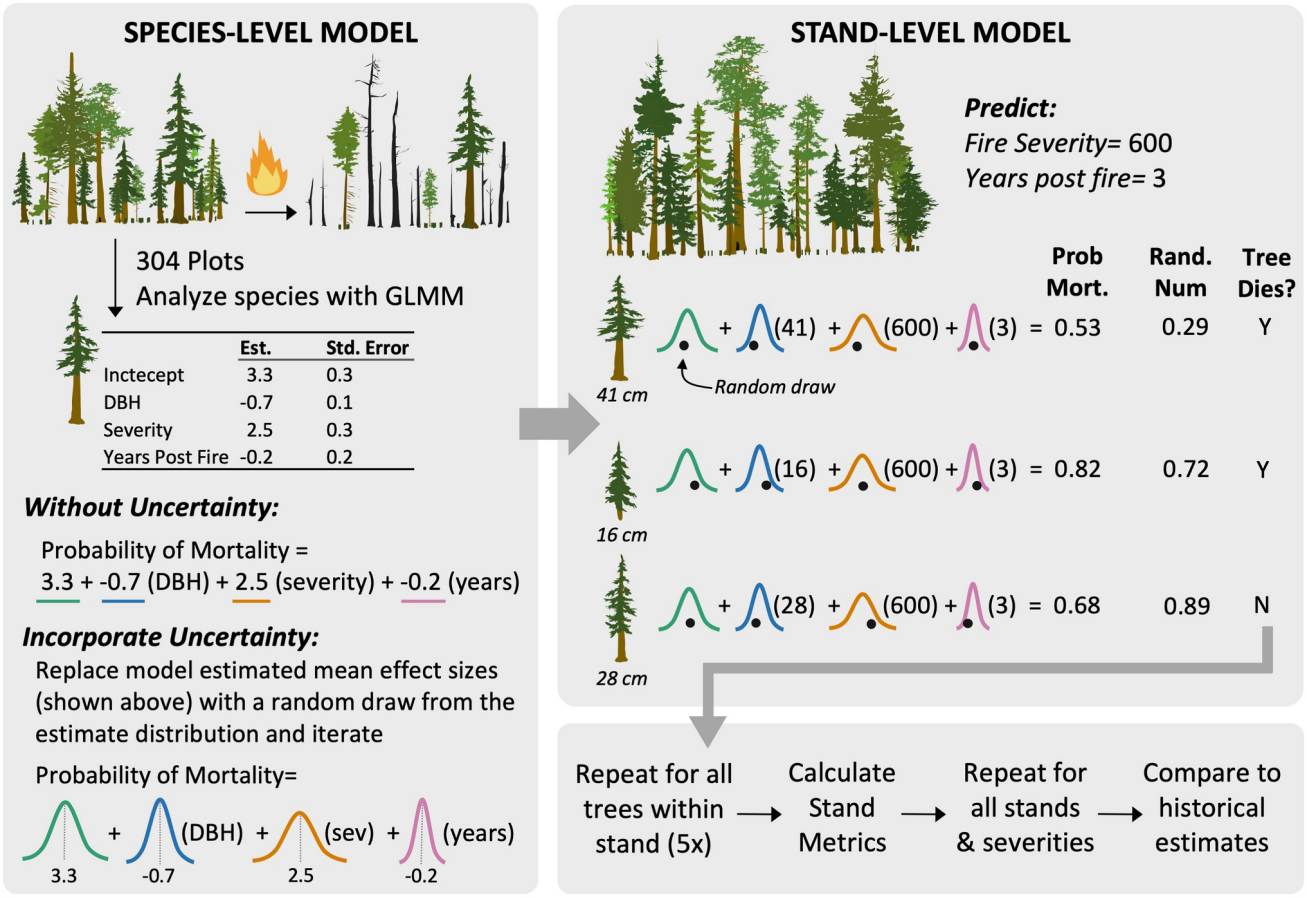
Conceptual diagram depicting simulation procedures. Left panel illustrates the development of a mortality model for one of the 24 individual species modeled and the Monte Carlo simulation framework employed to assess uncertainty in effect size estimates from Generalized Linear Mixed Models (GLMM). Top right panel illustrates application of species-level models to individual trees in contemporary stands (three example trees of the same species with different diameters displayed) within a Monte Carlo simulation framework. Probability of mortality for individual trees was calculated by replacing effect size point estimates with random values drawn from a normal distribution where the mean is the parameter estimate and the standard deviation is the standard error of the parameter from the GLMM equation. Estimates were then inverse logit transformed. This probability was compared to a random value generated from a uniform distribution between 0 and 1. If the randomly generated number was lower than the probability of mortality the tree was considered dead. The bottom right panel describes the iterative process used to assess stand-level model uncertainty and comparison of model estimates to historical estimates of forest conditions.

To predict post-fire stand structure and composition across a range of forests in the interior Pacific Northwest, we integrated datasets from remote sensing, field plots with pre- and post-fire measurements, contemporary forest inventories, and historical forest inventories and reconstructions in a novel modeling approach as shown in Fig 1. To incorporate and assess uncertainty of modeled estimates we used a Monte-Carlo simulation framework that carries estimates of uncertainty through modeling steps. First, we developed species-specific tree mortality models (hereafter “species-level models”) that relate probability of mortality to remotely sensed fire severity (RdNBR) and individual tree size from a field plot network in Oregon and Washington that experienced fire between measurement cycles. Next, we simulated fire by applying species-level models to individual trees within unburned stands in four National Forests in eastern Oregon to predict post-fire stand conditions across the observed range of RdNBR values (hereafter “stand-level models”). We then compared the results of simulated fire in contemporary stands to historical records and reconstructions of forest conditions to determine fire severity ranges that have the highest likelihood to restore historical conditions. Finally, we applied estimates from stand-level models to an example burned landscape to demonstrate the use of these methods for post-fire assessment and management (hereafter “landscape-scale model”).

We developed tree mortality models using a regional dataset and applied them to assess restorative fire severity ranges in eastern Oregon. The regional dataset primarily included plots from dry forest systems, but also included plots burned within fires in the Oregon Klamath Mountains, Oregon Cascades, and Washington North Cascades (Fig 1 in S1 Appendix). The eastern Oregon focal area is characterized by warm summers, cold winters, and precipitation falling mostly as snow [21]. Historically, fire-tolerant ponderosa pine was the dominant overstory species at lower elevations and co-occurred with western larch (*Larix occidentalis*) in northeastern Oregon. Less fire-tolerant white fir (*Abies concolor)*, grand fir (*Abies grandis*), and Douglas-fir (*Pseudotsuga menziesii*) occurred as components of mixed stands at higher elevations and in more mesic topographic settings [67]. Before Euro-American colonization, frequent, low severity fire (8–31-year return intervals) maintained forests conditions that were relatively resistant to fire, drought, and native pathogens [21, 68]. Fire exclusion due to decreased cultural burning, land use changes, and fire suppression policies has increased the abundance of less fire-tolerant species and overall forest density across these landscapes [21, 65].

### Species-level mortality models

To develop mortality models for 24 individual tree species, we used data from 22,419 trees recorded in 304 USDA Forest Service Region Six (Pacific Northwest) Current Vegetation Survey (CVS) plots that fell within 74 different fire perimeters (Tables 1 and 2 in S1 Appendix). Permanent CVS plots were systematically installed across all forested lands in Oregon and Washington between 1992 and 1997 and re-measured between 1997 and 2007 [69]. The subset of CVS plots used in these analyses were burned in wild or prescribed fires between measurement periods but had no other evidence of disturbance between sampling periods. Post-fire measurements were taken 0- to 13-years following fire. At each plot, data were recorded within five subplots each of which contained three nested, fixed-area plots [69]. Fire severity for each plot was estimated using the Relativized differenced Normalized Burn Ratio (RdNBR) [58]. RdNBR was calculated with the *LandTrendr* algorithm, which removes individual temporal variability resulting from measurements taken at different dates, using the pre-fire (year -1) and post-fire (year +1) time periods on a 90 x 90 m grid to approximate the CVS plot footprints. More details on the development and validation of burn severity maps are available in [6].

We modeled the probability of mortality for individual tree species as a function of diameter at breast height (DBH), fire severity (continuous RdNBR), and the number of years the tree was surveyed post-fire, to account for delayed mortality, using a generalized linear mixed model (GLMM) with a binomial distribution and logit link function [6, 70]. All models included plot as a random effect to account for within-plot spatial autocorrelation. Tree DBH ranged from 7 to 202 cm and fire severity ranged from -52 to 1,340 RdNBR (Table 2 in S1 Appendix). For species with greater than 2,000 observations, we compared two candidate models using AICc: one including the main effects of DBH, severity, years surveyed post fire, and an interaction between DBH and severity and a second including the main effects only. We selected models that included the interaction term for ponderosa pine and Douglas-fir given lower AICc values (ΔAICc = 5.5 and 5.5) and retained main-effects-only models for lodgepole pine (*Pinus contorta*), white fir, and tanoak (*Notholithocarpus densiflorus*) given a ΔAICc <2.

We calculated marginal and conditional R^2^ values for all models, on the logit link scale, to assess variation accounted for by fixed effects only and the whole model including random effects using the theoretical variances method described by [71]. Given variability in sample sizes between species (Table 2 in S1 Appendix), assessing significant effects solely with a p-value is problematic [72]. To compare the influence of different parameters on probability of tree mortality across species, we graphically display effect estimates from GLMMs using standardized data for each species, however analyses and modeling were performed using unstandardized data. Models were fit using the lme4 package (version 1.1.21), and model selection and R^2^ calculations were performed using the MuMIn package (version 1.42.6) in program R Version 3.6.1 [73–75].

Given that fire is a process with substantial inherent variability, we developed a modeling framework to explicitly incorporate variability in modeled estimates and explore how uncertainty is carried through the modeling process from individual- to stand- and landscape-scales (described below) using Monte Carlo simulation techniques [76]. To assess how variability around parameter estimates influenced the distribution of predicted tree mortality across the spectrum of observed burn severity, we iteratively calculated the conditional probability of mortality (i.e., population estimate conditional on the random effects) for an individual tree, three years post-fire, across the range of RdNBR fire severity values. For each iteration we replaced fixed effect parameter point estimates from the GLMM with simulated values (*β*_*MCx*_) generated by sampling from a normal distribution, N(μ, σ), where μ was set to the parameter estimate ${\hat{\beta }}_{x}$ and σ was set to the estimated standard error ${\hat{\sigma }}_{x}$ from the GLMM (Eq 1) [77]. Estimates of the probability of mortality ($\hat{\text{p}}$) were then calculated using the inverse-logit.


$$\begin{array}{l} logit\left(\hat{\text{p}}\left(Mort\right)\right)=\\ \left(\beta_{MC0}\sim N\left({\hat{\beta }}_{0},{\hat{\sigma }}_{0}\right)\right)+\left[\left(\beta_{MC1}\sim N\left({\hat{\beta }}_{1},{\hat{\sigma }}_{1}\right)\right)\times DBH\right]+\left[\left(\beta_{MC2}\sim N\left({\hat{\beta }}_{2},\;{\hat{\sigma }}_{2}\right)\right)\times Severity\right]+\\ \left[\left(\beta_{MC3}\sim N\left({\hat{\beta }}_{3},\;{\hat{\sigma }}_{3}\right)\right)\times Years\right]+\left[\left(\beta_{MC4}\sim N\left({\hat{\beta }}_{4},\;{\hat{\sigma }}_{4}\right)\right)\times DBH\times Severity\right] \end{array}$$ (1)

We graphically display model estimates for three DBH size classes per species, replicated 75 times for each RdNBR value (-50 to 1350, by 10) to build an output probability of mortality distribution. For models that included an interaction term we simulated estimates for DBH, severity, and the severity × size interaction independently. From the output distribution, we calculated the mean and standard deviation for each size along the range of RdNBR values and plotted these values using locally weighted scatterplot smoothing (LOESS) with first degree polynomial and span of 0.25. We display 10, 20, and 60 cm DBH size classes for conifers, and 10, 20, and 40 cm DBH for hardwoods. For species in which the largest size class was beyond the maximum DBH recorded in the dataset used to generate model estimates, we omit the largest size class. We report results for all 24 species for use in other landscapes or additional inquiry, even though not all were used in stand-level modeling described below.

**Fig 2:**
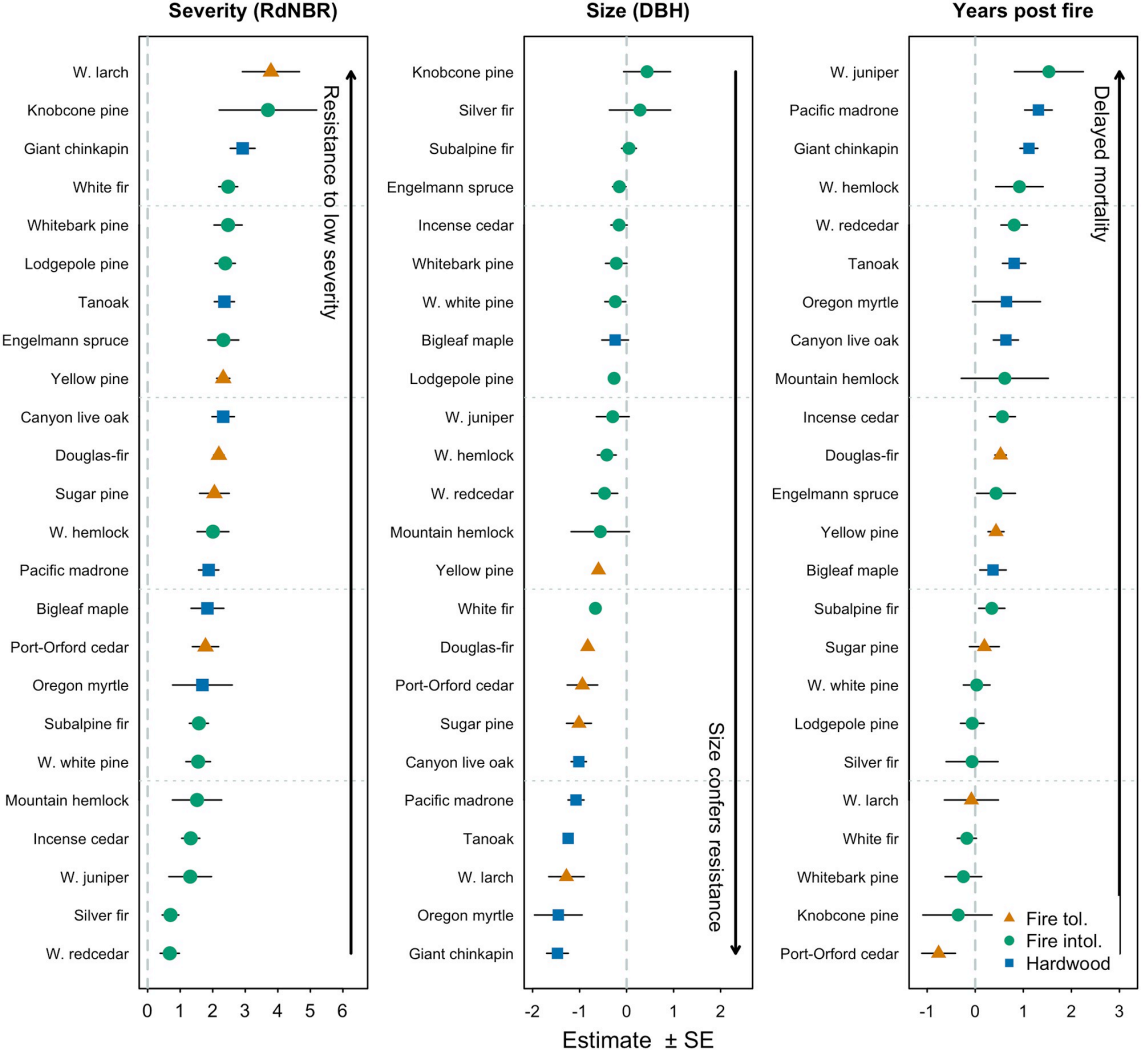
Standardized Generalized Linear Mixed Model coefficient estimates (± SE) for the effect size of fire severity, tree diameter at breast height, and number of years after the fire post-fire plot sampling occurred (Years Post Fire) on the probability of tree mortality for the 24 most common tree species from 304 forest inventory plots in Oregon and Washington that burned between initial measurements in 1992–1997 and remeasurements in 1997–2007. Standardized coefficients allow comparison between species models and predictors. For species in which the final selected model included a severity × size interaction term, plotted coefficients represent the effect of severity when size is held constant at its mean and vice versa. See Table 3 in S1 Appendix for unstandardized estimates and statistical results.

We compared model estimates from the full CVS plot dataset to models built using only the subset of CVS plots located in eastern Oregon to check for bias associated with applying regional estimates to stand-level modeling (described below). Estimates using the subset of CVS plots did not display appreciable effect size differences, so we comfortably utilized estimates from the full dataset in subsequent analyses (Fig 2 in S1 Appendix). For our analyses, we combined ponderosa pine and Jeffery pine (*Pinus jeffreyi*) to yellow pine and grand fir and white fir to white fir given highly similar ecologies, surveyor difficulties separating the species in the field and known hybridization [78]. We assigned species fire tolerances, used in graphical display and interpretation, based on classifications in [79, 80], and the Fire Effects Information System [81].

### Stand-level mortality models

To simulate stand-level residual tree density, basal area, and species composition following fire across a range of RdNBR severity values, we applied mortality probabilities from the species-level, unstandardized GLMM equations to individual trees in contemporary dry forest stands in eastern Oregon. We modeled stand structure and composition in inventoried, unburned stands on four National Forests across eastern Oregon classified as dry mixed-conifer or ponderosa pine forest types [68]. Stand-level data came from existing studies of contemporary, minimally managed stands representative of current forest conditions from the Malheur, Deschutes, Ochoco, and Fremont-Winema National Forests.

For the Malheur National Forest, we used plot data from 17 dry mixed-conifer stands and 18 ponderosa pine stands from areas without evidence of timber harvest from [67, 82]. We used data from 25 dry mixed-conifer stands and 11 ponderosa pine stands for the Deschutes National Forest and 9 dry mixed-conifer stands from the Ochoco National forests from a subset of the stands reported in [83] that did not display evidence of recent harvest or disturbance. For the Fremont-Winema National Forest we used 1998–2006 CVS plot data from 18 dry mixed-conifer and 18 ponderosa pine stands within the footprint of the former Klamath Reservation that displayed minimal evidence of recent timber harvest or disturbance. We expanded all stands to 1 ha using plot-specific expansion factors and removed all hardwood species and trees <15 cm to standardize across contemporary and historical stands (discussed below). For all datasets, we maintained biophysical groupings as originally published.

For each stand we assessed mortality from -50 to 1,000 RdNBR in 5-unit steps. We first estimated probability of mortality for each individual tree three years following fire, to account for delayed mortality, using the Monte Carlo simulation framework described above. To convert the probability of mortality for each tree to a binomial estimate of mortality, we compared each probability to a randomly generated value from a uniform distribution between zero and one [84, 85]. If the randomly generated number was lower than the probability of mortality, we considered the tree dead. Finally, we calculated live tree basal area, density, and proportion of the total stand basal area that consisted of yellow pine, Douglas-fir, white fir, western larch, and other species. We iterated this process 5 times for each stand to assess how uncertainty within the tree-level models propagated to the stand scale and graphically displayed stand basal area and density estimates from each simulation.

To evaluate similarity between modeled stands and historical structure and composition, we compared stand metrics following simulated fire mortality to three historical stand reconstruction datasets. For the Malheur National Forest we compared modeled estimates to a dendroecological reconstruction of forest conditions in the year 1880 using data first reported in [67, 82]. For the Fremont-Winema National Forest, we used historical forest conditions from a timber inventory collected between 1914 and 1922 on the former Klamath Reservation [86, 87]. For the Ochoco National Forest we used historical forest conditions from a timber inventory collected between 1922 and 1925 on the Warm Springs Indian Reservation [88, 89]. For stands on the Deschutes National Forest, we averaged estimates from the historical timber inventories on the Warm Springs Indian Reservation and former Klamath Reservation because plots used in this analysis on the Deschutes National Forest were geographically situated between the historical reconstructions [86]. Given differences between contemporary and historical datasets, we standardized datasets by expanding historical plots to 1 ha using plot-specific expansion factors, removing all hardwood species and trees <15 cm, and maintaining published biophysical settings as described above.

For the Malheur and Fremont-Winema forests, contemporary and historical plots were from overlapping geographic areas. For the Deschutes and Ochoco National Forests, we used the closest reliable historical estimates to our contemporary plot datasets. Substantial evidence suggests that historical conditions and fire regimes were not appreciably different between the locations of our contemporary plots and historical reconstructions. A recent regional review demonstrates that frequent low-severity fire regimes were ubiquitous across our study range [21]; [90] found similar historical fire return intervals, contemporary forest composition, and biophysical settings between the Ochoco National Forest and plots just south of the Warm Springs Indian Reservation; and historical inventories on the Warm Springs and former Klamath Reservations are remarkably similar and often applied to the Deschutes National Forest [86, 87, 91].

To assess fire severity ranges with the highest probability of restoring historical basal area and density across national forests and biophysical groups, we calculated the severity range in which ≥ 90%, ≥ 75%, and ≥ 50% of simulated stands fell within the historical range of variation (mean ± 1SD) for each location and forest type. To generalize the restorative fire severity range across all national forests and forest types modeled, we calculated the interquartile range (middle 50%) of severity values that fell within the ≥ 75% restorative range for basal area and density. We graphically display stand-level simulation results colored in relation to the proportion of simulations that fell within the range of variation (mean ± 1SD) of the geographically closest historical reconstruction.

To validate stand-level estimates, we used live tree data from an independent set of 44 one ha plots from the USDA Forest Service Pacific Northwest Research Station Annual Forest Inventory and Analysis program (FIA) PNW-FIA Integrated Database (IDB) (https://www.fs.usda.gov/pnw/tools/pnw-fiadb-forest-inventory-and-analysis-database). These plots burned in wildfires across dry forests in Oregon and Washington and were not included in the initial modeling. We selected plots that burned ≤ 15 years prior to measurement and did not display evidence of recent harvest (pre- or post-fire) or other disturbance. Given the limited number of plots that fit our criteria, we retained all plots within ponderosa pine or grand/white fir vegetation zone (Simpson vegetation map classification) [92], which was slightly broader than vegetation classifications used for the stand-level modeling.

We used three metrics to assess model performance. First, we assessed average prediction bias using Mean Bias Error calculated as the mean of the differences between observed FIA basal area and density and modeled mean basal area and density for the corresponding measured RdNBR, forest type, and nearest national forest. Next, we compared measured post-fire stand basal area and density for dry mixed conifer and ponderosa pine stands to 95% prediction intervals of our modeled estimates for the corresponding RdNBR, forest type, and nearest national forest. Confidence intervals from our stand-level model with lower bounds where the basal area was < 1 m^2^ ha^-1^ or density was <5 trees per hectare (TPH) were rounded down to include zero, given the larger plot size (1 ha) used for modeling inherently favored basal area or density estimates very close to but not truly zero at high fire severities. Finally, we assessed the proportion of times our model correctly predicted whether validation stands fell within the ≥ 75% restorative range for basal area or density for the corresponding RdNBR, forest type, and nearest national forest.

### Landscape-scale model

To demonstrate mapping restoration probabilities at the landscape scale and potential for this modeling to inform post-fire landscape restoration strategies, we applied our modeled restorative ranges for dry mixed conifer and ponderosa pine stands on the Malheur National Forest to the Parish Cabin fire, which burned 2,727 ha NE of Seneca Oregon in 2012. Pixels were assigned to restoration probability classes based on RdNBR values published in the MTBS database, ILAP Potential Vegetation Types (PVT; Integrated Landscape Assessment Project, inr.oregonstate.edu/ilap), and modeled restorative fire severity ranges for the Malheur National Forest. To assess post-fire forest composition and structure across severities and between forest types we calculated predicted residual stand basal area by species across tree size classes for several example locations within the Parish Cabin Fire. For this example, we did not filter landscape conditions beyond PVT, but if this method was operationalized, further place-based filtering (e.g., young forest, non-forest, or previous burns) may be warranted.

## Results

### Species-level simulations

Sample size for individual species in our dataset ranged from 94 for western juniper (*Juniperus occidentalis*) to 5,668 for Douglas-fir, roughly proportional to their occurrence in fire-prone forests of the Pacific Northwest (Table 2 in S1 Appendix). Accordingly, we have greater confidence in modeled estimates for more common species (n>2,000) including Douglas-fir (marginal R^2^ = 0.49), ponderosa pine (marginal R^2^ = 0.56), lodgepole pine (marginal R^2^ = 0.52), and white fir (marginal R^2^ = 0.56), which are the focus of our interpretation and use in stand-level modeling. These species account for 95% of individuals modeled in stand-level analyses.

**Fig 3:**
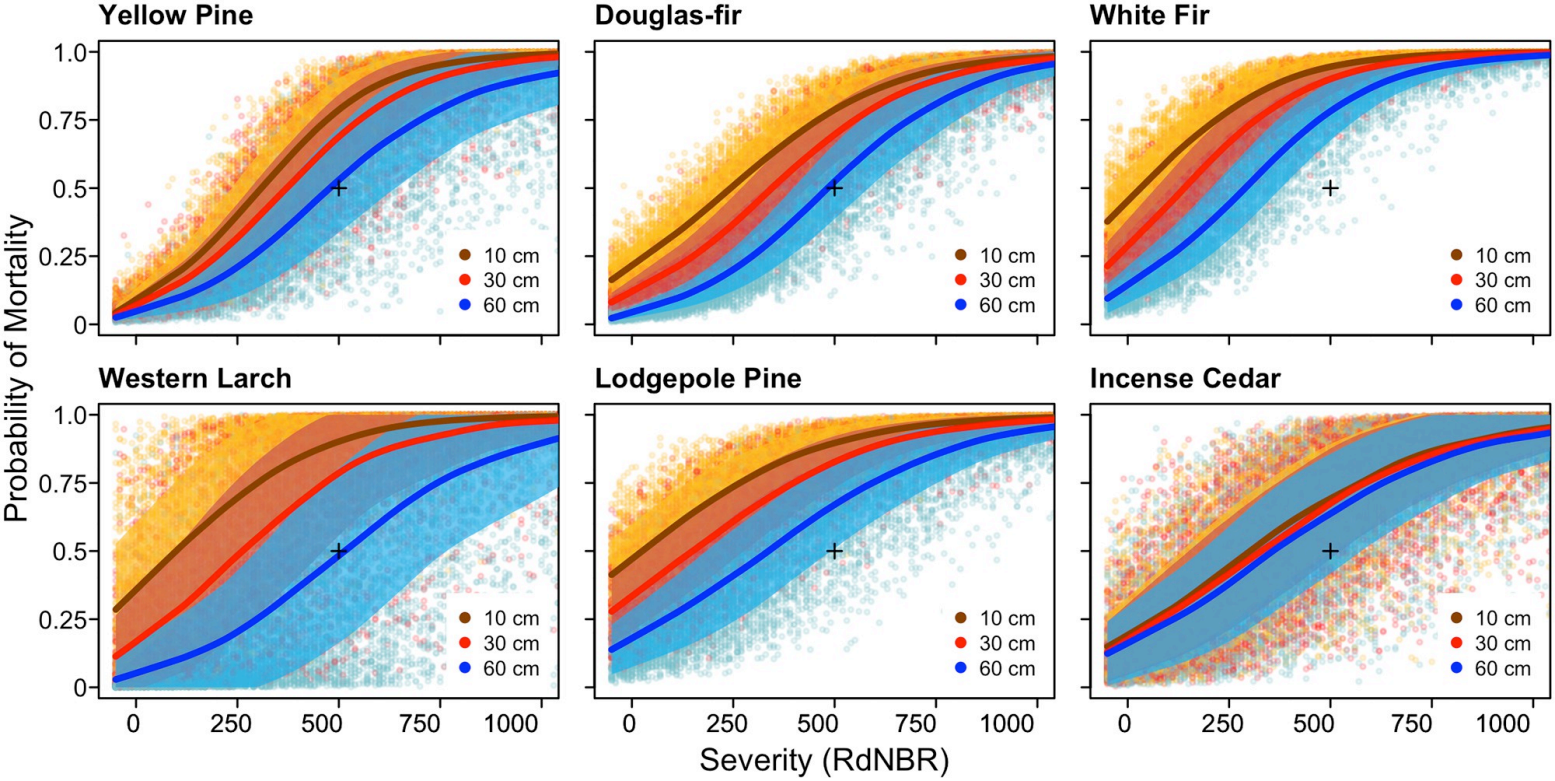
Probability of mortality for six common species in three size classes across the observed range of RdNBR burn severity. Points represent estimates from individual Monte Carlo Simulations. LOESS smoothed means and standard deviations are plotted for each size class. Plus sign placed at 0.5 probability of mortality and 500 RdNBR for reference. See Fig 3 in S1 Appendix for graphs of all modeled species.

Response of individual tree species to fire was generally consistent with published fire tolerances and functional traits, although our models highlighted several interesting mortality dynamics that are not reported by previous research (Figs 2 and 3). Predictably, all species displayed increased probability of mortality with increasing fire severity, however the strength of this effect varied by species, indicating differences in resistance to low- and moderate-severity fire (Figs 2 and 3, Fig 3 and Table 3 in S1 Appendix). The effect of fire severity alone—when DBH and years post fire are held constant at the mean for the species—did not clearly separate species commonly considered fire-tolerant or intolerant. However, the split between these species was much more evident in the effect of DBH, where trees classified as fire-tolerant display a strong effect of decreasing probability of mortality with increasing DBH (Fig 2) [79, 80].

Including the severity × size interaction term for Douglas-fir and yellow pine did not substantially alter predicted probability of mortality. The interaction term for yellow pine resulted in greater differentiation in probability of mortality between small and large diameter trees at high severities, whereas the interaction term for Douglas-fir resulted in greater differentiation between small and large diameter trees at low severities (Fig 3).

White fir probability of mortality, under contemporary conditions, was only slightly higher than ponderosa pine or Douglas-fir, which is inconsistent with the common assumption that grand and white fir are substantially less fire-tolerant (Figs 2 and 3) [79, 80]. We only assessed individuals ≥7 cm DBH, and seedlings or saplings of these species likely show substantial fire tolerance differences, which has important biological implications especially under frequent, low-severity fire regimes that were common historically. Notably, yellow pine and Douglas-fir were more vulnerable to delayed mortality than white fir (Fig 2).

**Fig 4:**
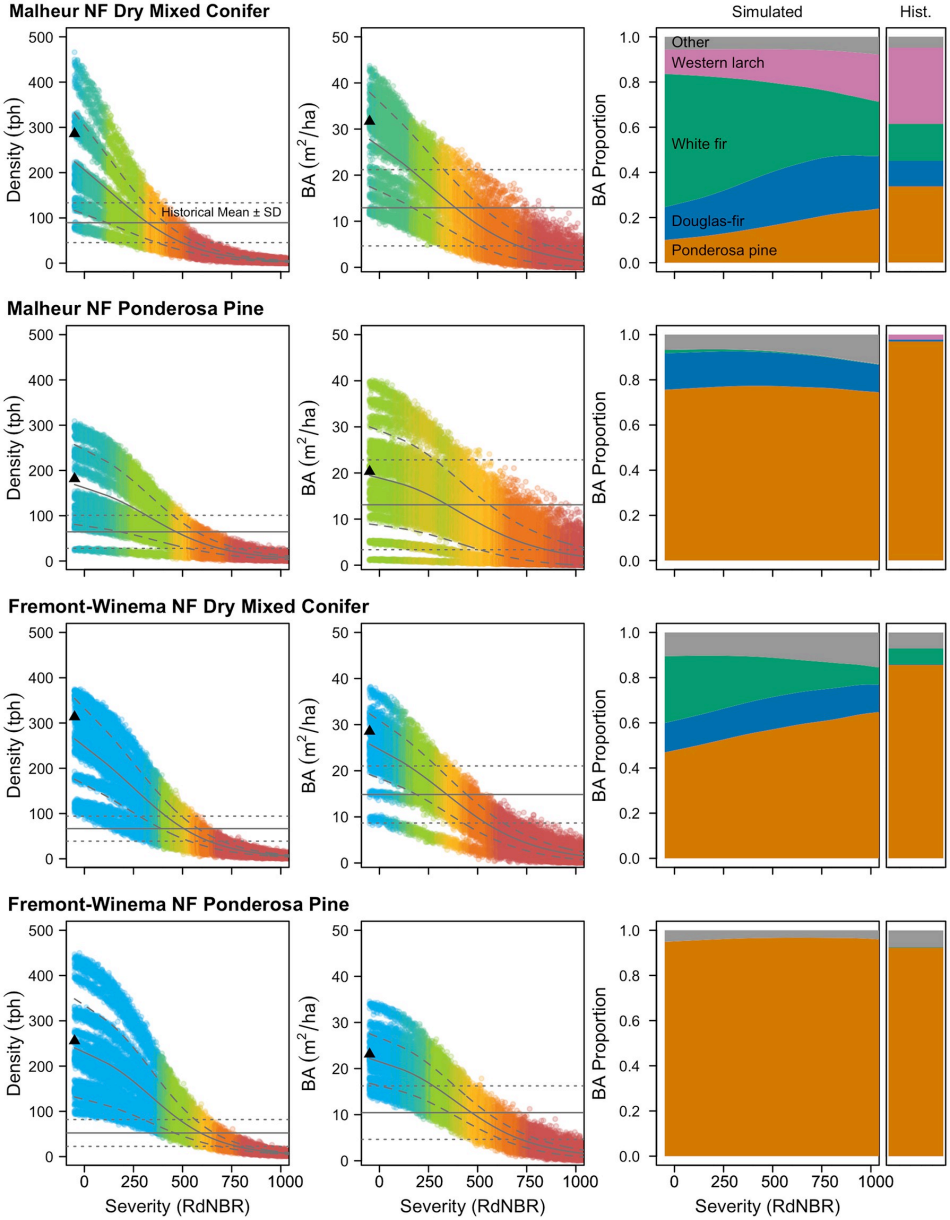
Density, basal area, and species composition of simulated dry mixed conifer and ponderosa pine stands in the Malheur and Fremont-Winema National Forests across a fire severity gradient from -50 to 1,000 RdNBR for trees ≥15cm. Mortality of individual trees within 17–18 stands was estimated 5 times across the fire severity gradient (in 5-unit steps) and stand density or basal area from each iterated stand are displayed as points. Points are colored according to the proportion that fall within the historical range of variation (mean ± 1SD, displayed as horizontal lines on each graph) reported for each forest type within each national forest with yellow representing the highest proportion of simulations that fell within the range. The loess smoothed mean ± 1SD of all simulations is displayed as an entire and dashed line within the simulated points. Triangles along the X-axis display unburned stand density and basal area. Basal area proportion is a loess smoothed average of all simulated stands for which total basal area was >0. Historical proportions are average basal area composition reported for the respective historical reconstructions. See Fig 4 in S1 Appendix for graphs for the Deschutes and Ochoco National Forest.

Lodgepole pine displayed some resistance to low-severity fire, but DBH had minimal influence on the probability of mortality or delayed mortality. Western larch and incense cedar (*Calocedrus decurrens*) had a relatively low sample sizes in our dataset (229 and 336 individuals, respectively), and were a relatively minor component of the tree community in our contemporary modeled plots but are species of ecological importance in many dry forests. In our dataset, western larch was the most fire-tolerant species, displaying strong resistance to low-severity fire and significantly lower mortality with increasing DBH. Given the small sample size for this species, and correspondingly wide confidence interval on effect size estimates there was substantial variation in probability of mortality when western larch was modeled in the Monte-Carlo framework, which propagated into our stand-level modeling (Figs 2–4, Figs 3 and 4 in S1 Appendix). Incense cedar is generally considered fire-intolerant [70, 81]; however, some studies have suggested mature individuals may be somewhat fire-resistant [48, 80, 93]. We did not detect any influence of tree DBH on probability of survival at mean fire severity for incense cedar, however these results should be interpreted cautiously given low sample size and the intermediate fire tolerance of the species (and others including western white cedar, *Thuja occidentalis*, and western white pine, *Pinus monticola*). Hardwood tree species, except for big leaf maple (*Acer macrophyllum*), consistently demonstrated a strong effect of DBH on the probability of mortality and were vulnerable to delayed mortality (Fig 2, Fig 3 in S1 Appendix).

### Stand-level simulations

**Fig 5:**
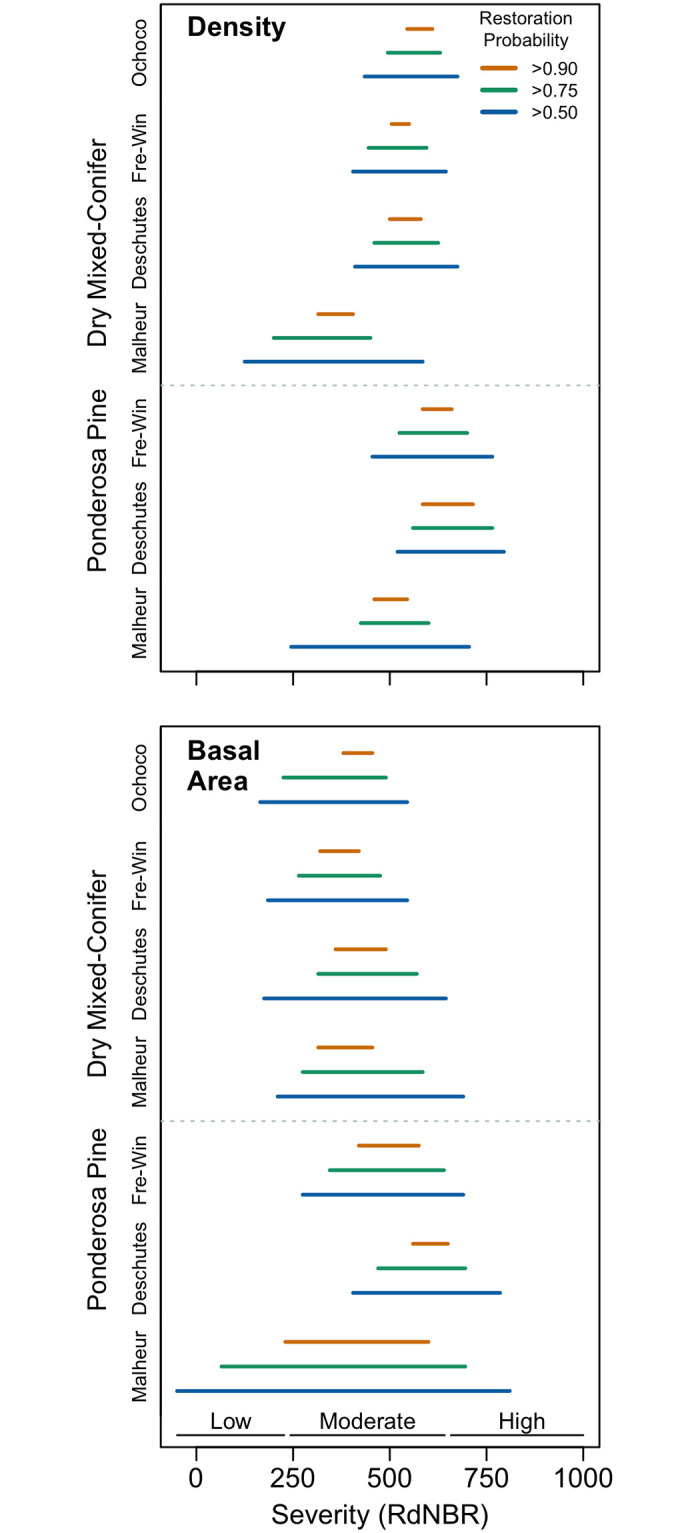
Fire severity ranges in which >0.9, >0.75, and >0.5 of the simulated stands were within the basal area or diameter historical range of variation (mean ± 1SD) for all national forests and forest types assessed. Fire severity classes included for reference from [6].

Prior to simulated burning, contemporary dry mixed conifer stand density and basal area were, on average, 4.7 and 2.3 times higher than average historical estimates and ponderosa pine stands were 4.6 and 2.3 times higher (Fig 4). Relatively consistent overall stand structural and compositional changes were observed for dry mixed conifer and ponderosa pine forest types in all four national forests across the fire severity gradient (Figs 4 and 5; Fig 4 in S1 Appendix). However, in all models, predicted post-fire stand metrics varied substantially at the stand-level, especially at lower fire severities, given diverse starting conditions and incorporation of estimate uncertainty (Fig 4, Fig 4 in S1 Appendix).

Dry mixed-conifer and ponderosa pine forests had similar target fire severity ranges for restoring historical tree density and basal area (Fig 5). In general, the restorative ranges for basal area and density were also similar, but the ranges for basal area were somewhat wider and encompassed a lower range of severities than those that restored historical density. The average range with a 90% probability of stand basal area restoration was 369–521 RdNBR, whereas the range for 75% probability of stand basal area restoration was 280 to 593 RdNBR (Fig 5). For reference, the observed fire severity range for plots used in our model development was -52 to 1,394 RdNBR, so these windows represent 10% and 22% of the observed range, respectively. The average range with a 90% probability of stand density restoration was 499–581 RdNBR and the range for 75% probability of restoration was 444 to 624 RdNBR, representing 6% and 12% of the observed range, respectively (Fig 5). Generally, severities between 365 and 560 RdNBR are most likely to restore basal area and density of ≥ 75% of dry mixed-conifer and ponderosa pine stands across the national forests studied. However, this range is on the lower end of the severity required to restore density in some forests, and basal area in some stands may be restored at lower severities.

In contrast, fire did not restore historical species compositions for dry mixed-conifer or most ponderosa pine forest types (Fig 4; Fig 4 in S1 Appendix). In dry mixed-conifer stands, moderate- and high-severity fire reduced the proportion of stand basal area comprised of Douglas-fir and white fir but did not restore historical dominance of yellow pine. Severities at which substantial declines of Douglas-fir and white fir began to occur were well above thresholds that would potentially restore overall stand density or basal area (Figs 4 and 5). Contemporary species composition was less departed in ponderosa pine stands, but fire did little to shift the composition (Fig 4). All model simulations displayed a slight increase in the average proportion of total basal in the other species category at high fire severities, which is an artifact of less common species having wider parameter effect size distributions leading to a wider range of randomly selected effect sizes in Monte Carlo simulations.

**Fig 6:**
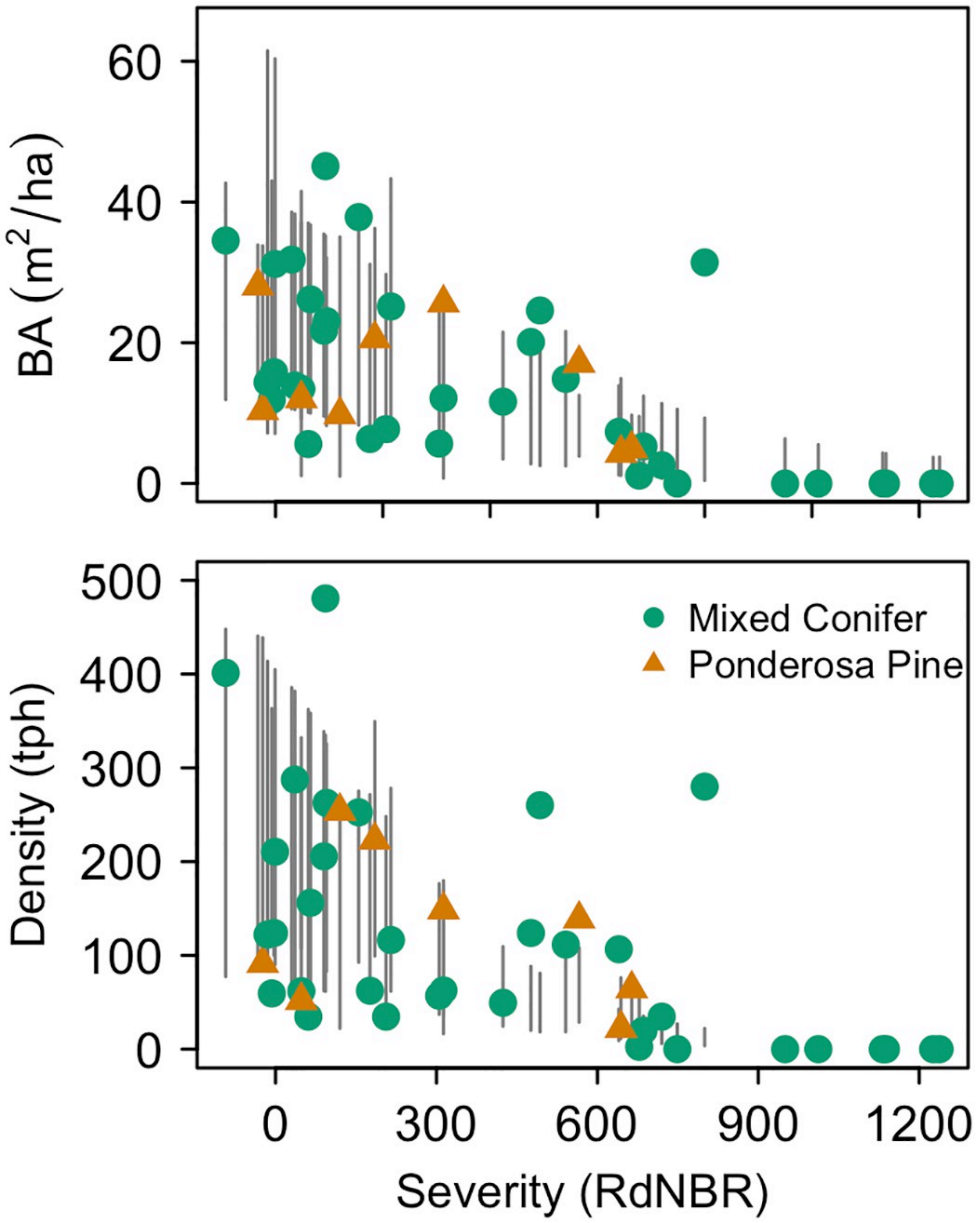
Stand basal area and density of plots used for model validation. Points for each plot are displayed on a vertical line representing the 95% confidence interval of modeled estimates for the nearest national forest and corresponding forest type and RdNBR.

Mean Bias Error calculations demonstrated that our model, on average, overestimated basal area by 0.68 m^2^/ha and underestimated stand density by 17.67 trees/ha. Basal area of 80% of validation plots fell within our simulated 95% confidence interval, while 64% of plots fell within the 95% confidence interval for stand density (Fig 6). The model correctly predicted whether validation plots fell within the ≥ 75% restorative range 77% of the time for stand density and 64% of the time for basal area.

### Landscape-scale

**Fig 7:**
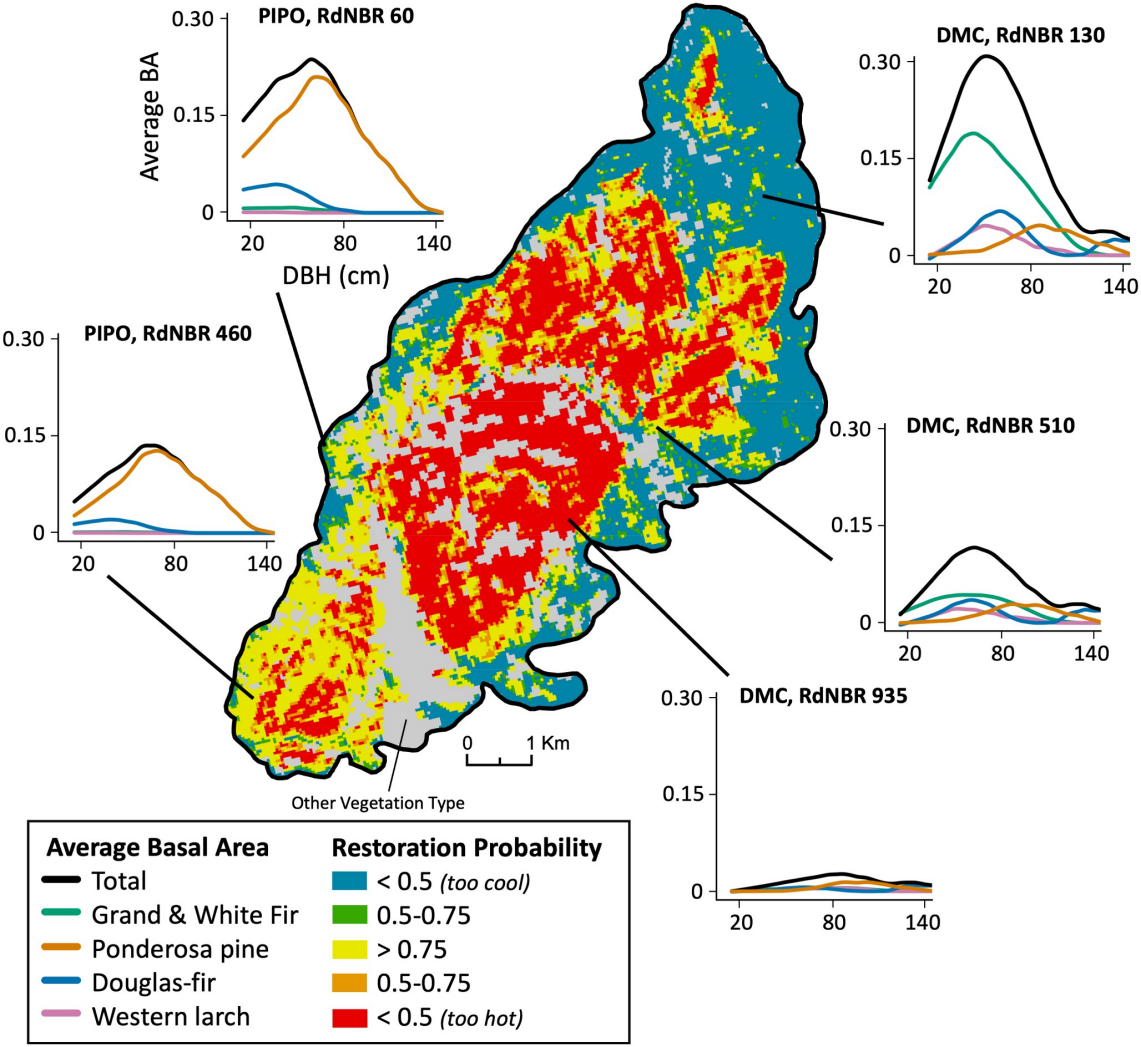
RdNBR fire severity for the 2012 Parish Cabin fire on the Malheur National Forest reclassified according to the probability of restoring stand basal area. Cool colors represent pixels with RdNBR values below the severity range with the highest restoration probability and warm colors represent pixels with RdNBR values above this range. Graphs display model estimated average basal area distribution within a 1 ha stand, by tree diameter for all trees ≥15 cm and major species at select RdNBR burn severities observed on the map for ponderosa pine (PIPO, left) and dry mixed conifer (DMC, right) forest types. Grey areas on map indicate other vegetation types.

Restorative fire severity ranges mapped for the Parish Cabin fire serve as a landscape-scale case study to examine locations where wildfire was most likely to restore historical basal area given measured fire severity and mapped vegetation types (Fig 7). Within the Parish Cabin fire 25% of pixels had a basal area restoration probability of ≥ 0.75 and 37% had ≥ 0.50 probability of restoration. Of the 62% of pixels that had a restoration probability of <0.50, 31% burned too hot and 31% burned too cool. Ponderosa pine stands had a substantially higher proportion of pixels (78%) with a ≥ 0.50 probability of basal area restoration than dry mixed-conifer pixels (28%).

As fire severity increased, the basal area of small trees decreased, and large trees represented a greater proportion of the total stand basal area for both the ponderosa pine and dry mixed-conifer forest types (Fig 7). In ponderosa pine stands, large trees almost entirely consist of ponderosa pine, whereas in dry mixed-conifer stands, large tree basal area consists of ponderosa pine, Douglas-fir, and, to some degree, white fir, and western larch. At high severities the few remaining trees tend to be ponderosa pine or very large Douglas-fir.

## Discussion

Based on our evaluation of contemporary fire severity and its relationship to changes in tree density, basal area, and forest composition, we demonstrate that single fire events are unlikely to restore resistant and resilient forest structure and composition in contemporary dry forests in the interior Pacific Northwest at the stand-scale (Fig 4). Across a broad geographic range, low- and moderate-severity fire did not kill enough large, established fire-intolerant trees (white fir and Douglas-fir) to restore historical forest species composition that resulted from frequent, interacting low-severity fires. These results illustrate the imprecise nature of fire alone as a restoration tool and need to consider synergies between wildfire effects and active forest management for restoration efforts [1, 38, 94, 95].

Across four National Forests, including multiple physiographic provinces and forest types, we found strikingly similar patterns of stand structural and compositional effects from fire (Fig 5). A relatively narrow range of moderate fire severities, roughly 365–560 RdNBR, were most likely to restore stand density and basal area, aligning with findings from several smaller-scale field-based studies of fire effects [18, 47, 53, 59, 96]. However, stand composition, which is a critical component of forest restoration and resistance to future disturbance, remained departed from historical conditions even at high fire severities (Fig 4) [1, 89, 97]. Composition of dry mixed-conifer stands was more departed from historical composition than ponderosa pine stands, but even at very high fire severities ponderosa pine stands still contained substantially more fire-intolerant species than were historically present (Fig 4; Fig 4 in S1 Appendix).

White fir is commonly defined as a fire-intolerant species, however we found relatively high survival of larger white fir at low and moderate fire severities (Fig 3), and that fire severities hot enough to kill a substantial proportion of the large white fir in a stand will also kill a substantial proportion of large ponderosa pine, western larch, and Douglas-fir (Figs 2 and 3) [79, 80]. This relatively high white fir survival supports the premise that historical species composition in these forests was primarily maintained by mortality of fire-intolerant seedlings or saplings in frequent, low-severity fires rather than direct fire mortality of large, established individuals [21, 47, 67, 84, 98]. Without additional active management, large white fir are likely to persist and potentially increase future dominance in many burned stands given the species’ prolific reseeding capacity [49, 99–102]. Large trees are ecologically important, however there are key differences between large white fir and large ponderosa pine including crown characteristics, rooting depth, drought tolerance, heartwood proportion, and longevity, which can have substantial ecological impacts [21, 68, 84]. In areas where restoration of large/old trees and associated forest structure is prioritized over species composition targets, the ability of large white fir to survive low and moderate severity fires may broaden restorative fire severity windows and achieve restoration goals.

Restorative windows for basal area and density were very similar for dry mixed-conifer and ponderosa pine forest types, which aligns with work suggesting dry forests across the interior Pacific Northwest all burned frequently and compositional differences were driven by biophysical differences (Fig 5) [21, 103–105]. The primary difference we detected between the two forest types was a smaller departure from historical species compositions in ponderosa pine stands, leading to stand compositions that more closely resembled historical conditions within fire severity zones with the highest basal area/density restoration probabilities (Fig 4, Fig 4 in S1 Appendix). Although fire could not restore species composition in our simulations, it did increase mean tree size for both forest types, which is consistent with historical conditions and should increase stand resistance to future fire (Fig 7) [67, 87, 89, 101].

### Fire as a process in dry forests

These results highlight the role of fire as a recurrent disturbance process in dry forests that shapes ecosystem structure and function over time through repeated and interacting events rather than a series of discrete disturbance and recovery events [19, 52, 106]. Given these forests develop with repeated fire events rather than simply following single fire events, reintroduction of a single fire following a protracted disturbance-free interval cannot fully restore ecosystem structure and functioning [21, 90, 107]. Fire effects are realized from the scale of each tree (e.g. pruning of lower branches, wounding, or mortality) to patch (e.g. competition, contagious process spread) to landscape (e.g. patch mosaics, refugia) across a range of fire intervals and severities [1, 101, 106, 108]. However, in systems operating under recurrent disturbances, the effects from individual disturbances are strongly influenced and modulated by the impacts of past events [109, 110].

The characteristics that emerge from frequent and interacting fires often do not exist in the absence of recurrent disturbance and legacies of frequent-fire regimes are rapidly declining in contemporary, unburned landscapes [16, 51, 65, 87, 111, 112]. Intervention to re-initiate and restore these processes often requires a sequence of treatments over time including mechanical thinning, off-season managed wildfire, cultural burning, and/or prescribed fire [53, 84, 96, 113]. However, most contemporary wildfire events are typically managed as discrete disturbance events rather than part of an ongoing process [14, 20, 114]. This is in stark contrast to Indigenous fire stewards who have managed fire for millennia as a repeated disturbance process to enhance ecosystem resilience, reduce community fire risk, and promote important, foods, fibers, and medicines across landscapes [29, 115–119].

### Model uncertainty and potential sources of error

The Monte-Carlo simulation framework we developed allows scientists and managers to evaluate uncertainty in modeled processes and visualize the full range of potential outcomes (Figs 3 and 4) [120]. This uncertainty exists in the large majority of fire effects analyses given limited sample size and the many complex and interacting factors that drive fire behavior and effects, but it is often ignored even when used in predictive capacities [55, 56, 121].

We focused our modeling of restorative fire severity on dry forest systems because we had robust species-level data for the dominant species in dry mixed conifer and ponderosa pine PVTs and model uncertainty was greater for species with small samples sizes from less fire-prone landscapes (Figs 2 and 3; Fig 3 in S1 Appendix). We feel our stand-level results can reasonably be extrapolated to mature, dry forests across the interior Pacific Northwest, but care should be taken when extrapolating beyond this domain especially within systems characterized by mixed or high severity fire regimes where spatial patterns of fire severity are highly important [19, 79]. Care should also be taken when extrapolating to reburned or previously thinned stands given challenges interpreting RdNBR values between stands with dramatically different pre-fire conditions [122, 123]. Interpretation of tree-level results should account for sample size variation for different species. New data should be available within the next several years to strengthen the tree-level and stand-level estimates as recently burned long-term inventory plots are remeasured.

Our stand-level modeling did not include trees <15 cm because we did not have historical data for smaller trees, however smaller trees are abundant in many contemporary forests and the relationship between small tree mortality and fire severity is an important avenue for future inquiry. We excluded all plots from this modeling that displayed any evidence of disturbance other than fire to minimize error in our estimates of delayed mortality, however, these estimates should be considered in conjunction with existing evidence for species of interest. The variability within our modeling framework contracted substantially at the stand level, and forest-level variance predominantly stemmed from variable pre-fire stand conditions, rather than variability in estimates for individual trees (Fig 4; Fig 4 in S1 Appendix).

While we endeavored to transparently carry uncertainty through our modeling process, we were not able to characterize the uncertainty of including remotely sensed severity metrics, model selection, treatment of random effects, and biases associated with excluding recently disturbed sites in our stand-level modeling. Spatial distance between contemporary plots and historical forest inventories may contribute uncertainty to restorative fire severity zones for the Ochoco and Deschutes National Forests. However, given consistent results across all four National Forests and extensive evidence for widespread frequent, low-severity fire regimes, this is unlikely [21, 87, 90].

### Management implications

Several post-fire restoration frameworks have recently been proposed to improve management of recently burned landscapes in the western United States, which describe general pathways to evaluate post-fire landscapes, determine likely future trajectories, and prioritize treatments [38, 124, 125]. This study provides a quantitative tool for post-fire landscape evaluation by reclassifying commonly used maps of RdNBR fire severity classes to probability of restoration classes, which provides the opportunity for managers and researchers to link forest restoration goals and wall-to-wall maps of predicted post-fire condition that are readily available (Fig 7) [66]. These methods allow managers and researchers to develop specific restoration treatments based on amount and spatial distribution of areas that are likely close to or below restoration targets, areas that burned ‘too hot’, and allocation of limited post-fire resources [126, 127].

Areas where fire largely restored historical basal area and density can potentially be transitioned to an active treatment maintenance schedule to gradually regain historical, and likely more fire- and drought- tolerant, species composition and stand structure [20, 33, 39, 41, 97, 128, 129]. Restoration opportunity may be even greater in stands that burned slightly too cool to fully reduce basal area or density because fire likely removed many of the smaller, fire-intolerant trees and the opportunity remains to use mechanical thinning to further move species composition towards desired conditions (Fig 7) [18, 68]. Management in areas that burned at high severity and surpassed desired restoration windows likely depends on resultant patch size and could include replanting in areas where regeneration failure is likely or edge hardening smaller patches to reduce risk of subsequent high-severity fire [43, 124, 125].

All results from large-scale models need to be interpreted through an understanding of local knowledge of landscape and site conditions and history [130]. For instance, high-graded stands without pre-fire structure and composition that will allow for restoration might benefit from slightly higher fire severity accompanied by planting of target species. As with all treatments in fire-prone ecosystems, treatment maintenance and work towards reintroduction of fire as a process is critical for the restoration of resilience, as is demonstrated by the near universal failure of fire, at any severity, to restore both stand structure and composition in our models [43, 95, 101, 131].

Narrow stand structure or composition targets often do not represent historical conditions across large landscapes [32, 37, 101]. Therefore, transitioning landscapes from current fire regimes with uncharacteristically large proportions of high-severity fire to fire regimes dominated by repeated, low-severity fire events will not be achieved by universally applying rigid and uniform treatments [21, 68, 101]. Our iterative Monte-Carlo simulation approach demonstrates a general range of conditions in which fire has restorative benefits but does not represent hard targets for all locations. These windows of restoration potential are intended to aid in strategic planning and identify areas of benefit and opportunity that can be further filtered, refined, or expanded based on local landscape conditions, management objectives, and desired forest heterogeneity [13, 20].

Historically, low-severity fire was extensive in dry forests, but small patches of high-severity fire (typically <0.5 ha and rarely >10 ha) occurred as well and this heterogeneity should be not be discounted [19, 21, 89, 102, 132]. Early seral and non-forest conditions also play an important role in the landscape ecology of fire and are a key ecological resource [101, 133]. Future work integrating the fine-scale structural and compositional patterns we explore in this work with analysis of larger landscape-scale patterns, such as analyses presented by [38], would be powerful [124]. Historical conditions may not always represent desired modern restoration targets for a variety of reasons, including contemporary and future climate, management objectives, or habitat for threatened and endangered species [31, 134, 135]. While we used historical conditions for this study, target zones could easily be adjusted or expanded based on desired future targets.

## Conclusions

Although there is a growing acceptance of the fundamental role of fire in many ecosystems, post-wildfire assessment and management in the western United States is overwhelmingly focused on negative impacts of wildfire events [20, 91, 114, 131]. Remotely sensed metrics of fire severity are often viewed as a measure of ‘degree of harm’ rather than a ‘degree of benefit’, and management following fire most often focuses on restoring or rehabilitating areas most negatively affected. There is a growing body of research exploring the restorative potential of wildfire and increasing discussion among the wildfire management community on the intersection of forest restoration objectives and wildfire response [14, 18, 20, 33, 42]. Managed wildfire, landscape-scale prescribed fire, and cultural burning can efficiently treat large areas. However, our work demonstrates that in historically frequent-fire forests, contemporary, moderate-severity fire can restore tree density or basal area, but often does not restore composition and the patterns and processes resulting from fire as an ongoing ecological process [38]. Quantifying and mapping where wildfires were the most beneficial can help focus post-fire management to better align with broader restoration objectives and identify areas where active management, such as mechanical thinning, planting, or burning, may be the most valuable.

## Supporting information

S1 Appendix Supplemental results. Supplemental species- and stand-level model inputs, numerical outputs, and graphical results.
(PDF)
