# Supplementary material for: Too hot, too cold, or just right: Can wildfire restore dry forests of the interior Pacific Northwest?
Source: PLoS One. 2023 Feb 27;18(2):e0281927. doi: 10.1371/journal.pone.0281927 (PMC9970105; doi:10.1371/journal.pone.0281927)
Supplement: S1 Appendix — Supplemental species- and stand-level model inputs, numerical outputs, and graphical results. (PDF) [file pone.0281927.s001.pdf]

Appendix. Greenler, S. M., Dunn, C. D, Johnston, J. D., Reilly, M. J., Merschel, A. G., Hagmann, R. K., Bailey, J. D. (2022). Too hot, too cold, or just right: Can wildfire restore dry forests of the interior Pacific Northwest? *PLOSOne.*

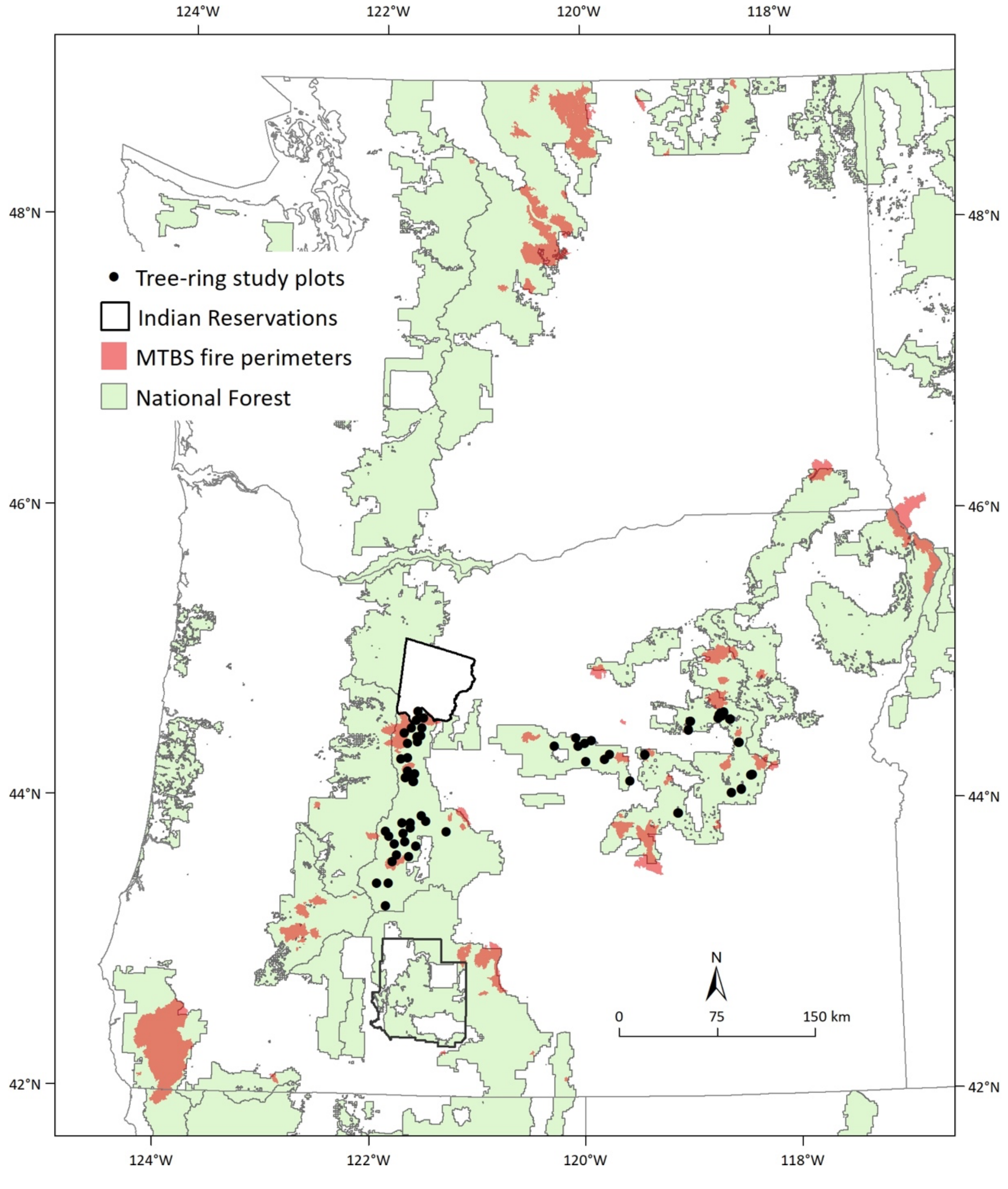

Figure 1: Species-level mortality models were developed from Current Vegetation Survey (CVS) plots falling within the MTBS fire perimeters shown above and listed in Appendix S1: Tables S1, S2. Stand-level mortality models were developed from the tree-ring study plots indicated above and from CVS plots falling within the boundary of the former Klamath Reservation, now the Fremont-Winema National Forest. Historical structure and composition were derived from tree-ring study plots within the Malheur National Forest and from 1914-1924 timber inventories conducted on the Warm Springs and Klamath Indian Reservations. State

boundaries courtesy of the U.S. Geological Survey. National Forest boundaries courtesy of the USDA Forest Service. Boundaries for the Warm Spring Indian Reservation and former Klamath Reservation courtesy of the Confederated Tribes of Warm Springs and The Klamath Tribes, respectively. Fire perimeter data courtesy of the USDA Forest Service and U.S. Geological Survey Monitoring Trends in Burn Severity (MTBS) project.

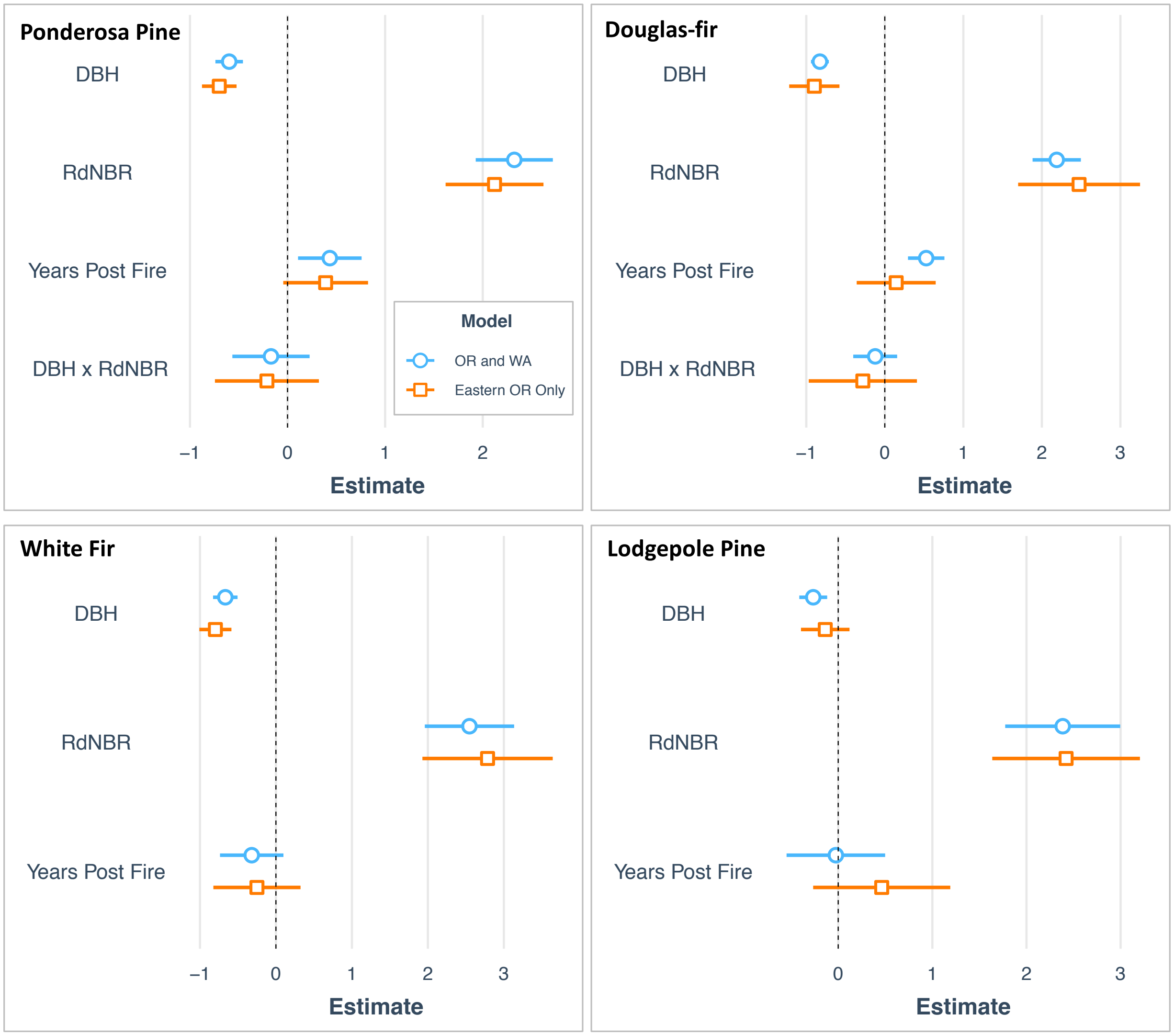

Figure 2. Comparison of standardized effect size estimates and 95% confidence intervals for species-level mortality models run with the full set of CVS plots and subset of CVS plots from eastern Oregon where estimates were applied for stand-level modeling.

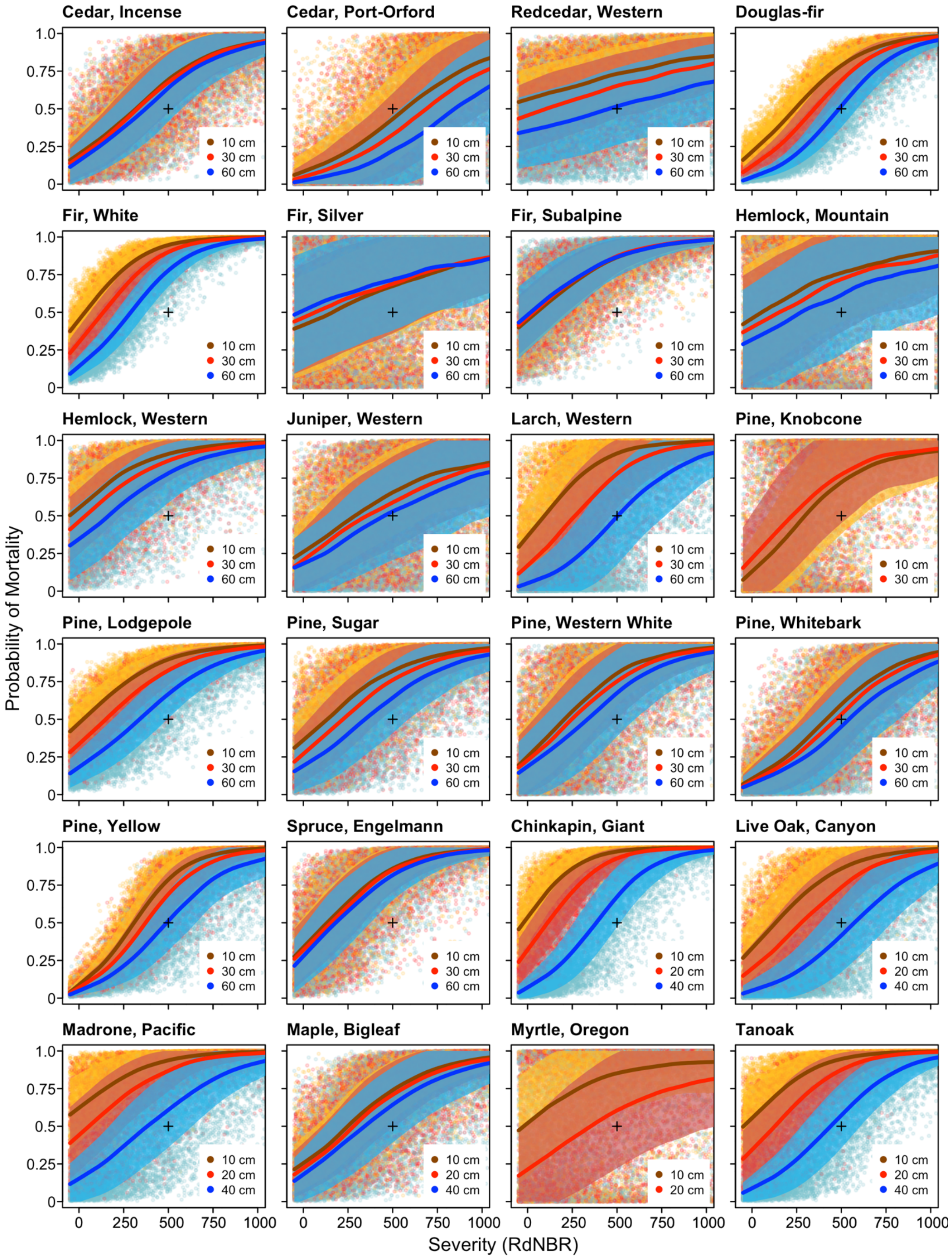

Figure 3. Probability of mortality for 24 common tree species across Oregon and Washington in three size classes across the observed range of RdNBR burn severity. Points represent estimates from individual Monte Carlo Simulations and LOESS smoothed means and standard deviations are plotted for each size class. Plus sign placed at 0.5 probability of mortality and 500 RdNBR for reference.

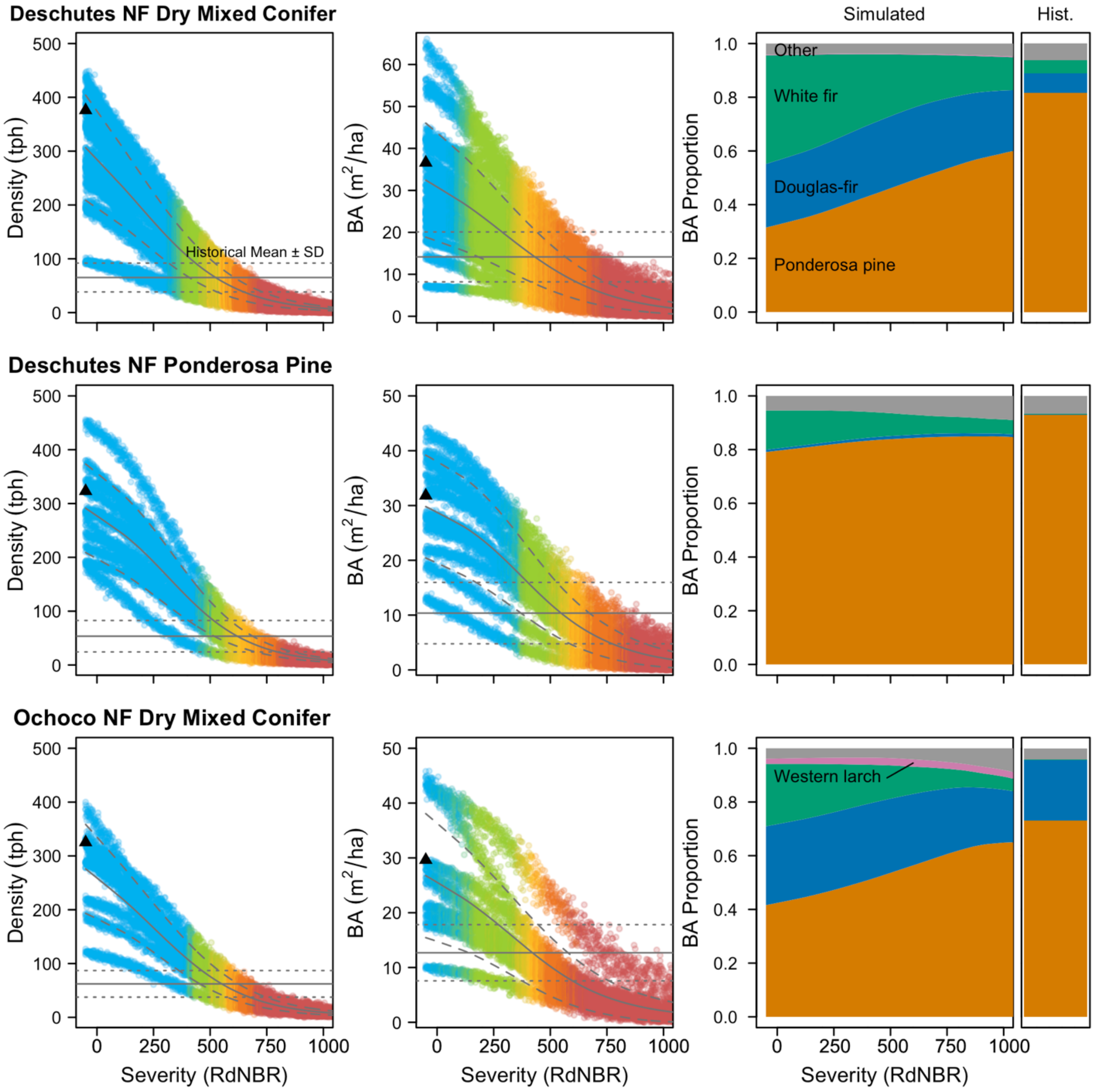

Figure 4. Density, basal area, and composition of simulated dry mixed conifer and ponderosa pine stands in the Deschutes and Ochoco National Forests across a fire severity gradient from -50 to 1,000 RdNBR for trees ≥15cm. Mortality of individual trees within 9-25 stands was estimated 5 times was across the fire severity gradient (in 5-unit steps) and stand density or basal area from each iterated stand are displayed as points. Points are colored according to the proportion that fall within the historical range of variation (mean ± 1SD, displayed as horizontal lines on each graph) reported for each forest type within each national forest with yellow representing the highest proportion of simulations that fell within the range. The loess smoothed mean ± 1SD of all simulations is displayed as an entire and dashed line within the simulated points. Triangles along the X-axis display unburned stand density and basal area. Basal area proportion is a loess smoothed average of all simulated stands for which total basal area was >0. Historical proportions are average basal area composition reported for each respective historical reconstruction.

Table 1. Fire and plot survey dates for the 74 different fires within Oregon and Washington used to model tree mortality.

Fire Name	Fire Date	Pre-Fire Surveys	Post-Fire Surveys
Blossom Complex (Mendenhall)	1994	1993	2003
Boundary	1994	1994	2003-2005
Jordan Springs	1994	1994	2001-2007
Little Malheur Complex (Ironside)	1994	1993-1994	2000-2003
Reed	1994	1994	2004
Thunder	1994	1994	2004
Tyee Creek	1994	1994	2002
Alder Ridge	1996	1996	1999
Bull Complex (Bull)	1996	1994	1999
Bull Complex (Summit)	1996	1994-1995	1999-2007
Moolack Complex (Charlton)	1996	1993-1995	1999-2002
Salt Complex (Salt Creek)	1996	1994	2003
Skeleton-Evans West (Evans West)	1996	1994	2001-2007
Skeleton-Evans West(Skeleton)	1996	1993	2001
Sloan's Ridge	1996	1994-1995	2000-2003
Spring	1996	1994-1995	2004
Tower	1996	1993-1994	1999-2005
Wheeler Point	1996	1994	1999-2005
North 25	1998	1995-1996	1999-2002
2063	1999	1995	2000
Repeater	1999	1993-1995	1999-2003
Thomas	1999	1995	1999
W D Lake	1999	1995	2000-2004
Eastside Complex (Deep Creek)	2000	1995	2004
Hash Rock	2000	1994	2003
Maloney Creek	2000	1994	2003
Rocky Hull	2000	1997	2007
Fourth Of July	2001	1996	2006
Indian Springs	2001	1993	2002
Lakeview Complex (South Warner)	2001	1994	2002
Libby South	2001	1996	2007
Mt. Leona Complex	2001	1995-1996	2003-2006
Quartz	2001	1995	2003
Rex Creek Complex (Rex Creek)	2001	1995-1997	2002-2006
Thirty Mile	2001	1995-1997	2004-2007
Virginia Lake Complex (Bailey Mountain)	2001	1994	2004
747 Complex (747)	2002	1994	2003-2006
Acker	2002	1995	2004
Apple	2002	1994-1997	2004-2007

Big Bend	2002	1995-1996	2004-2007
Biscuit Complex (Biscuit)	2002	1993-1997	2003-2005
Boulder	2002	1995-1997	2004-2007
Deer Point	2002	1995-1997	2002-2006
Easy	2002	1994-1995	2004-2007
Eyerly Complex (Eyerly)	2002	1994-1995	2004-2007
Flagtail	2002	1994	2004-2007
Grizzly Complex (Silver)	2002	1994-1996	2002-2005
Grizzly Complex (Winter)	2002	1994-1996	2002-2005
Monument-Malheur Complex (Monument)	2002	1993-1995	2003-2006
Quartz Mt. Complex (Middle Mountain)	2002	1994	2004-2005
Quartz Mt. Complex (Quartz Mountain)	2002	1994	2004
Roberts Creek	2002	1994	2004
Tiller Complex (Tallow)	2002	1995	2004
Tool Box Complex (Tool Box)	2002	1994-1996	2002-2005
18 Fire	2003	1993	2004-2007
B&B Complex (Booth)	2003	1993-1995	2004-2007
Clark	2003	1997	2005
Davis	2003	1994-1995	2004-2007
Fawn Peak Complex (Farewell)	2003	1994-1996	2004-2007
Isabel	2003	1994-1995	2004-2007
Kelsay Complex (Kelsay)	2003	1996	2007
Link	2003	1995	2004
Mineral Park	2003	1995	2004
Needles	2003	1993-1994	2004-2007
Togo Mountain	2003	1994-1996	2003-2006
Fischer	2004	1996	2006
Pot Peak-Sisi Ridge Complex	2004	1997	2006
Sycan	2004	1996	2005
School	2005	1995	2005
Cascade Crest Complex (Black Crater)	2006	1994	2007
Thorn Creek	2006	1994	2007
Tripod Complex (Spur Peak)	2006	1995-1997	2007
Tripod Complex (Tripod)	2006	1995-1996	2007
Egley Complex (Egley)	2007	1995-1996	2007

Table 2. Summary statistics for the 24 tree species used in analyses including the total sample size; number of plots and fires the species was recorded on; diameter at breast height (DBH, cm); RdNBR burn severity (Relative differenced Normalized Ratio); and the range of years elapsed between fire and mortality measurements.

Species	Common Name	Sample Size	Plot Occur.	Fires Occur.	DBH			RdNBR			Yrs. Post Fire Range
					Mean (SD)	Median	Range	Mean (SD)	Median	Range	
Abies amabilis	Silver Fir	165	6	4	26.7 (16.3)	24.6	7.6-82.8	1041.6 (171.6)	1048.3	194.5-1255.7	1-4
Abies grandis & concolor	Grand & White Fir	2059	95	37	28.3 (19.8)	22.1	7.6-140	622.3 (372.4)	555.2	-30.7-1256.6	0-11
Abies lasiocarpa	Subalpine Fir	1157	55	23	21.8 (13.5)	16.8	7.6-84.6	924.1 (320.8)	1079.0	-7.9-1393.9	0-11
Acer macrophyllum	Bigleaf Maple	114	23	9	22.1 (12.9)	18.0	7.6-69.1	340.4 (332.3)	226.6	37-999.3	0-8
Arbutus menziesii	Pacific Madrone	565	57	6	24.3 (11.6)	22.1	7.6-64.5	462.4 (331.4)	298.0	37.5-1207.6	0-5
Calocedrus decurrens	Incense Cedar	336	48	14	29.7 (26.6)	18.7	7.6-182.9	614.7 (241.9)	636.4	60.8-1207.6	0-9
Chamaecyparis lawsoniana	Port-Orford Cedar	113	12	1	38.3 (29)	33.3	7.6-128.3	679.2 (300.5)	829.6	60.6-969.5	1-3
Chrysolepis chrysophylla	Giant Chinkapin	356	37	5	20.9 (13.9)	16.1	7.6-80	496.4 (368.8)	488.0	37.5-1207.6	1-5
Juniperus occidentalis	Western Juniper	94	20	14	26.5 (18.1)	18.9	7.6-83.8	392.9 (261.4)	284.2	-6.9-919.8	0-13
Larix occidentalis	Western Larch	229	38	20	38 (18.7)	37.1	7.6-104.6	516.5 (447.6)	379.4	-52-1256.6	0-11
Lithocarpus densiflorus	Tanoak	2145	50	2	19.8 (12.2)	15.5	7.6-113.3	436.3 (369.7)	296.1	37-1170.2	0-4
Picea engelmannii	Engelmann Spruce	1110	52	23	33.3 (21.6)	33.4	7.6-126.2	871.2 (361.2)	951.8	-52-1393.9	0-11
Pinus albicaulis	Whitebark Pine	233	18	10	25.5 (13.1)	22.1	7.9-81.8	702 (391)	730.3	-7.9-1213.4	1-6
Pinus attenuata	Knobcone Pine	248	23	2	19.7 (9.9)	16.1	7.6-50	797.9 (294.8)	956.7	42-1021.1	1-4
Pinus contorta	Lodgepole Pine	2501	93	33	16.6 (8.6)	14.2	7.6-77.5	654.9 (474)	528.6	-52-1393.9	0-11
Pinus lambertiana	Sugar Pine	302	55	8	61 (44.8)	49.5	7.6-176.3	643.2 (383.4)	648.3	-11.4-1207.6	1-9
Pinus monticola	Western White Pine	461	34	11	23 (14)	18.0	7.6-79.2	752.5 (214.9)	829.6	3.2-1238.2	1-11
Pinus ponderosa & jeffreyi	Ponderosa & Jeffery Pine	2578	138	54	34.2 (23.8)	30.2	7.6-142.5	448.8 (339.5)	405.0	-52-1238.2	0-13
Pseudotsuga menziesii	Douglas-fir	5668	220	51	43 (30.5)	37.8	7.6-202.9	500.3 (357.3)	417.8	-52-1393.9	0-11
Quercus chrysolepis	Canyon Live Oak	851	38	2	17.6 (11.1)	13.5	7.6-64	507.9 (371.9)	432.7	37-1170.2	1-4
Thuja plicata	Western Redcedar	99	9	4	35.1 (20.2)	37.1	7.9-91.4	378.8 (359.1)	219.7	3.2-1135.7	1-5
Tsuga heterophylla	Western Hemlock	464	17	8	29.3 (18.4)	26.0	7.6-110.2	624.1 (383.7)	526.3	3.2-1135.7	1-8
Tsuga mertensiana	Mountain Hemlock	460	8	4	35.3 (17.3)	37.0	7.6-91.2	1009.4 (219.3)	1048.3	194.5-1255.7	1-6
Umbellularia californica	Oregon Myrtle	111	18	2	11.9 (4.9)	10.2	7.6-35.8	339.8 (216.9)	387.3	37-999.3	0-4

Table 3. Unstandardized regression coefficients, error estimates, and p-values for the effect of fire severity (RdNBR), tree diameter at breast height (DBH, cm), number of years after the fire post-fire plot sampling occurred (Years Post Fire), and RdNBR × DBH for select models on the probability of tree mortality for the 24 most common tree species from 304 forest inventory plots in Oregon and Washington that burned between initial measurements in 1992-1997 and remeasurements in 1997-2007. Marginal and conditional R^2 values were calculated using theoretical variances for the logit link scale. ***= P<0.001, **=p<0.01, *= p,0.05

Species	Intercept	DBH	RdNBR	Yrs. Post Fire	SD Rand Eff.	RdNBR × DBH	Marginal R^2	Conditional R^2
Abies amabilis	-0.694 (2.154)	0.017 (0.04)	0.004 (0.001)***	-0.044 (0.362)	0.000		0.17	0.17
Abies grandis & concolor	0.294 (0.43)	-0.033 (0.004)***	0.007 (0.001)***	-0.064 (0.073)	1.419		0.56	0.73
Abies lasiocarpa	-0.748 (0.828)	0.004 (0.012)	0.005 (0.001)***	0.152 (0.12)	1.389		0.34	0.59
Acer macrophyllum	-1.888 (0.865)*	-0.019 (0.022)	0.006 (0.001)***	0.238 (0.177)	0.654		0.49	0.55
Arbutus menziesii	-2.417 (0.824)***	-0.093 (0.015)***	0.006 (0.001)***	1.316 (0.286)***	1.345		0.51	0.68
Calocedrus decurrens	-2.614 (0.866)***	-0.006 (0.007)	0.005 (0.001)***	0.332 (0.157)*	1.190		0.32	0.52
Chamaecyparis lawsoniana	-0.191 (1.012)	-0.032 (0.011)***	0.006 (0.001)***	-0.955 (0.442)*	0.524		0.56	0.59
Chrysolepis chrysophylla	-2.033 (0.557)***	-0.105 (0.017)***	0.008 (0.001)***	1.083 (0.18)***	0.000		0.77	0.77
Juniperus occidentalis	-2.465 (1.33)	-0.016 (0.019)	0.005 (0.003)*	0.375 (0.175)*	1.731		0.37	0.67
Larix occidentalis	-0.071 (1.342)	-0.068 (0.02)***	0.008 (0.002)***	-0.026 (0.183)	2.433		0.60	0.86
Lithocarpus densiflorus	-1.421 (0.627)*	-0.102 (0.008)***	0.006 (0.001)***	0.89 (0.267)***	1.432		0.58	0.74
Picea engelmannii	-1.225 (1.037)	-0.007 (0.007)	0.006 (0.001)***	0.15 (0.14)	2.183		0.42	0.76
Pinus albicaulis	-1.958 (0.916)*	-0.017 (0.017)	0.006 (0.001)***	-0.163 (0.252)	0.000		0.68	0.68
Pinus attenuata	-3.086 (1.33)*	0.044 (0.05)	0.013 (0.005)**	-0.373 (0.779)	0.000		0.79	0.79
Pinus contorta	0.226 (0.508)	-0.031 (0.009)***	0.005 (0.001)***	-0.019 (0.074)	1.459		0.52	0.71
Pinus lambertiana	-0.864 (0.97)	-0.023 (0.006)***	0.005 (0.001)***	0.124 (0.202)	1.652		0.49	0.72
Pinus monticola	-1.413 (1.402)	-0.017 (0.016)	0.007 (0.002)***	0.027 (0.247)	1.762		0.28	0.63
Pinus ponderosa & jeffreyi	-2.71 (0.481)***	-0.014 (0.005)***	0.008 (0.001)***	0.139 (0.057)**	1.489	-0.00003 (0.00002)*	0.56	0.74
Pseudotsuga menziesii	-1.856 (0.372)***	-0.035 (0.003)***	0.005 (0.001)***	0.286 (0.065)***	1.619	0.00002 (0.00001)*	0.49	0.72
Quercus chrysolepis	-1.931 (0.782)**	-0.091 (0.015)***	0.006 (0.001)***	0.665 (0.271)**	1.145		0.55	0.68

Thuja plicata	-1.062 (0.864)	-0.023 (0.014)**	0.002 (0.001)*	0.528 (0.179)***	0.000	0.39	0.39
Tsuga heterophylla	-0.468 (0.928)	-0.023 (0.011)**	0.005 (0.001)***	0.32 (0.172)	1.018	0.42	0.56
Tsuga mertensiana	-1.095 (3.01)	-0.032 (0.036)	0.007 (0.003)*	0.343 (0.506)	1.636	0.29	0.61
Umbellularia californica	1.43 (1.654)	-0.294 (0.103)***	0.008 (0.004)	0.556 (0.604)	1.863	0.45	0.73
